# Hemophagocytic Lymphohistiocytosis Syndrome Associated with Gaucher Disease Type 2

**DOI:** 10.4274/tjh.2014.0037

**Published:** 2014-09-05

**Authors:** Bahoush Gholamreza, Miri-Aliabad Ghasem

**Affiliations:** 1 Iran University of Medical Sciences, Ali Asghar Children’s Hospital, Pediatric Hematology-Oncology, Tehran, Iran; 2 Zahedan University of Medical Sciences, Children and Adolescent Health Research Center, Department of Pediatric Hematology-Oncology, Zahedan, Iran

**Keywords:** Gaucher disease, Hemophagocytic lymphohistiocytosis, Hepatosplenomegaly

## TO THE EDITOR

**Figure 1:**
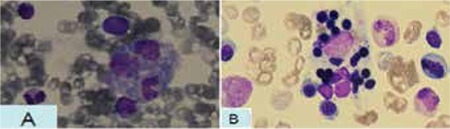
A) A macrophage that has phagocytosed a band cell and a lymphocyte B) A macrophage that has engulfed many normoblast.

We present the case of a 5.5-month-old female infant hospitalized because of fever, lethargy, pallor, poor feeding, convulsion, and developmental delay. She was the first child of the family and her parents were relatives. Physical examination revealed hepatosplenomegaly, opisthotonus, and pitting edema. Laboratory studies showed pancytopenia (WBC: 3500/mm3, Hb: 6 g/dL, Plt: 40.000/mm3), coagulopathy (PT: 19.4 s, aPTT: 41 s, fibrin degradation products: 1.8 mg/mL, D-dimer: 4 mg/L), AST of 132 U/L, ALT of 107 U/L, ALP of 313 U/L, triglycerides of 280 mg/dL, and albumin of 2.7 g/dL. Analysis of the cerebrospinal fluid (CSF) revealed lymphocytic pleocytosis. Serum ferritin level was 1020 ng/mL and fibrinogen level was 150 mg/dL. Other test results, including renal function tests, electrolytes, serum and urine amino acid chromatography, TORCH study, and virology studies (HIV, HCV, HBV, HAV, EBV), were normal. Blood, urine, stool, CSF, and bone marrow cultures were all negative. Bone marrow examination revealed hemophagocytosis caused by macrophages (Figure 1). It also showed an increase in the number of histiocytic cells, which was not typical of Gaucher disease. However, dried blood spot testing showed a drastic reduction of 8.45 pmol/spot (normal range: 200-2000 pmol/spot in 20 h) in the beta-glucosidase enzyme activity and confirmed the diagnosis of Gaucher disease. Since 6 of the 8 criteria of hemophagocytic lymphohistiocytosis (HLH) syndrome were observed in the patient, she was subjected to treatment with the HLH 2004 Protocol [2], broad-spectrum antibiotics, and anticonvulsant drugs while supportive measures were also taken. Despite the aforementioned efforts, the patient’s clinical condition gradually worsened and she died 10 days after the beginning of the treatment. It was not possible to study the mutations associated with primary HLH or to measure the level of sIL2R and the activity of NK cells. Informed consent was obtained.

This case is important because it is an example of HLH syndrome associated with Gaucher disease type 2. Gaucher disease is a lysosomal storage disorder that is caused by the deficiency of beta-glucocerebrosidase enzyme. Gaucher disease has 3 types. Type 2, which is known as the acute infantile form, is characterized with neurological symptoms and death in early childhood [1]. HLH syndrome is a rare and rapidly progressive disease that has a primary (familial) and a secondary (acquired) form. If this disorder is left untreated, it can quickly lead to death [2]. Diagnosis of primary HLH is confirmed by studying the related genetic mutations, such as STX11, UNC13D, and PRF1. The secondary type of HLH also develops after infections or with rheumatologic diseases, Chediak–Higashi syndrome, Griscelli syndrome, X-linked lymphoproliferative disease, malignancies, and inborn errors of metabolism. This potentially fatal disease is caused by uncontrolled over-production of inflammatory cytokines by cytotoxic T lymphocytes and histiocytes [2,3]. HLH syndrome is rarely associated with Gaucher disease. It was Lee et al. that first found erythrophagocytosis in Gaucher cells [4]. Hibbs et al. [5] and Bitton et al. [6] also reported erythrophagocytic activity in Gaucher cells. In another study, similar clinical symptoms were seen in patients with Gaucher disease type 2 and patients with HLH [7]. Approximately 15% of Gaucher cells were found to have abnormal morphologies or significant erythrophagocytic activity [8]. Sharpe et al. reported a female infant with Gaucher disease type 2 manifesting as severe HLH [9]. Only that study addressed the association of these 2 diseases, while other reports have only found erythrophagocytosis in Gaucher cells.

Although it was not possible to study the genetic mutations of HLH in this patient, on the basis of the familial background of her parents, the progressive trend of her disease, and no response of disease to treatment, it can be concluded that the patient possibly suffered from primary HLH. Measurement of the activity of beta-glucosidase enzyme confirmed the diagnosis of Gaucher disease. Due to the presence of severe and rapidly progressive neurologic symptoms, the patient was diagnosed with Gaucher disease type 2. These 2 diseases have many overlapping clinical symptoms. Therefore, in similar occasions, a careful evaluation of the presence of storage diseases such as Gaucher disease is recommended. 

## CONFLICT OF INTEREST STATEMENT

The authors of this paper have no conflicts of interest, including specific financial interests, relationships, and/ or affiliations relevant to the subject matter or materials included.
